# Deep COVID DeteCT: an international experience on COVID-19 lung detection and prognosis using chest CT

**DOI:** 10.1038/s41746-020-00369-1

**Published:** 2021-01-29

**Authors:** Edward H. Lee, Jimmy Zheng, Errol Colak, Maryam Mohammadzadeh, Golnaz Houshmand, Nicholas Bevins, Felipe Kitamura, Emre Altinmakas, Eduardo Pontes Reis, Jae-Kwang Kim, Chad Klochko, Michelle Han, Sadegh Moradian, Ali Mohammadzadeh, Hashem Sharifian, Hassan Hashemi, Kavous Firouznia, Hossien Ghanaati, Masoumeh Gity, Hakan Doğan, Hojjat Salehinejad, Henrique Alves, Jayne Seekins, Nitamar Abdala, Çetin Atasoy, Hamidreza Pouraliakbar, Majid Maleki, S. Simon Wong, Kristen W. Yeom

**Affiliations:** 1 Department of Radiology, School of Medicine, Stanford University, Stanford, CA 94305 USA; 2 Unity Health Toronto, University of Toronto, Toronto, ON M5S Canada; 3 Division of Radiology, Amir Alam Hospital, Tehran University of Medical Sciences, Tehran, Iran; 4 Rajaie Cardiovascular Medical and Research Center, Iran University of Medical Sciences, Tehran, Iran; 5 Henry Ford Health System, Detroit, Michigan USA; 6 Universidade Federal de São Paulo (UNIFESP), São Paulo, Brazil; 7 Department of Radiology, Koç University School of Medicine, Istanbul, Turkey; 8 Hospital Israelita Albert Einstein, São Paulo, Brazil; 9 Department of Radiology, School of Medicine, Kyungpook National University, Daegu, Korea; 10 School of Medicine, Tehran University of Medical Sciences, Tehran, Iran; 11 Advanced Diagnostic and Interventional Radiology Research Center(ADIR), Medical Imaging Center, Imam Khomeini Hospital Complex, Tehran University of Medical Sciences, Tehran, Iran; 12 Department of Electrical Engineering, Stanford University, Stanford, CA 94305 USA

**Keywords:** Radiography, Viral infection, Computational science

## Abstract

The Coronavirus disease 2019 (COVID-19) presents open questions in how we clinically diagnose and assess disease course. Recently, chest computed tomography (CT) has shown utility for COVID-19 diagnosis. In this study, we developed Deep COVID DeteCT (DCD), a deep learning convolutional neural network (CNN) that uses the entire chest CT volume to automatically predict COVID-19 (COVID+) from non-COVID-19 (COVID−) pneumonia and normal controls. We discuss training strategies and differences in performance across 13 international institutions and 8 countries. The inclusion of non-China sites in training significantly improved classification performance with area under the curve (AUCs) and accuracies above 0.8 on most test sites. Furthermore, using available follow-up scans, we investigate methods to track patient disease course and predict prognosis.

## Introduction

Coronavirus disease 2019 (COVID-19) caused by severe acute respiratory syndrome corona virus 2 (SAR-Cov-2) has inflicted a global health crisis and was declared a pandemic in March 2020^1^. The high transmission rates that can lead to respiratory distress and multiple organ failures, requisite critical care resources, and rising mortality^2–4^ have prompted an urgent need for early detection, accurate diagnosis, and predictive tools.

Real-time reverse-transcription polymerase chain reaction (RT-PCR) is the primary method for SAR-Cov-2 diagnosis. However, RT-PCR has shown variable sensitivity and specificity^5–7^ either due to insufficient viral load, sample collection methods, or lack of definitive reference standards^8,9^. Studies have reported characteristic imaging features of COVID-19 pneumonia^10^ and proposed chest CT to either complement RT-PCR or serve as the initial workup in highly suspected cases given the potential for false negative RT-PCR^11–13^ and to gauge disease severity^14,15^.

**Fig. 1:**
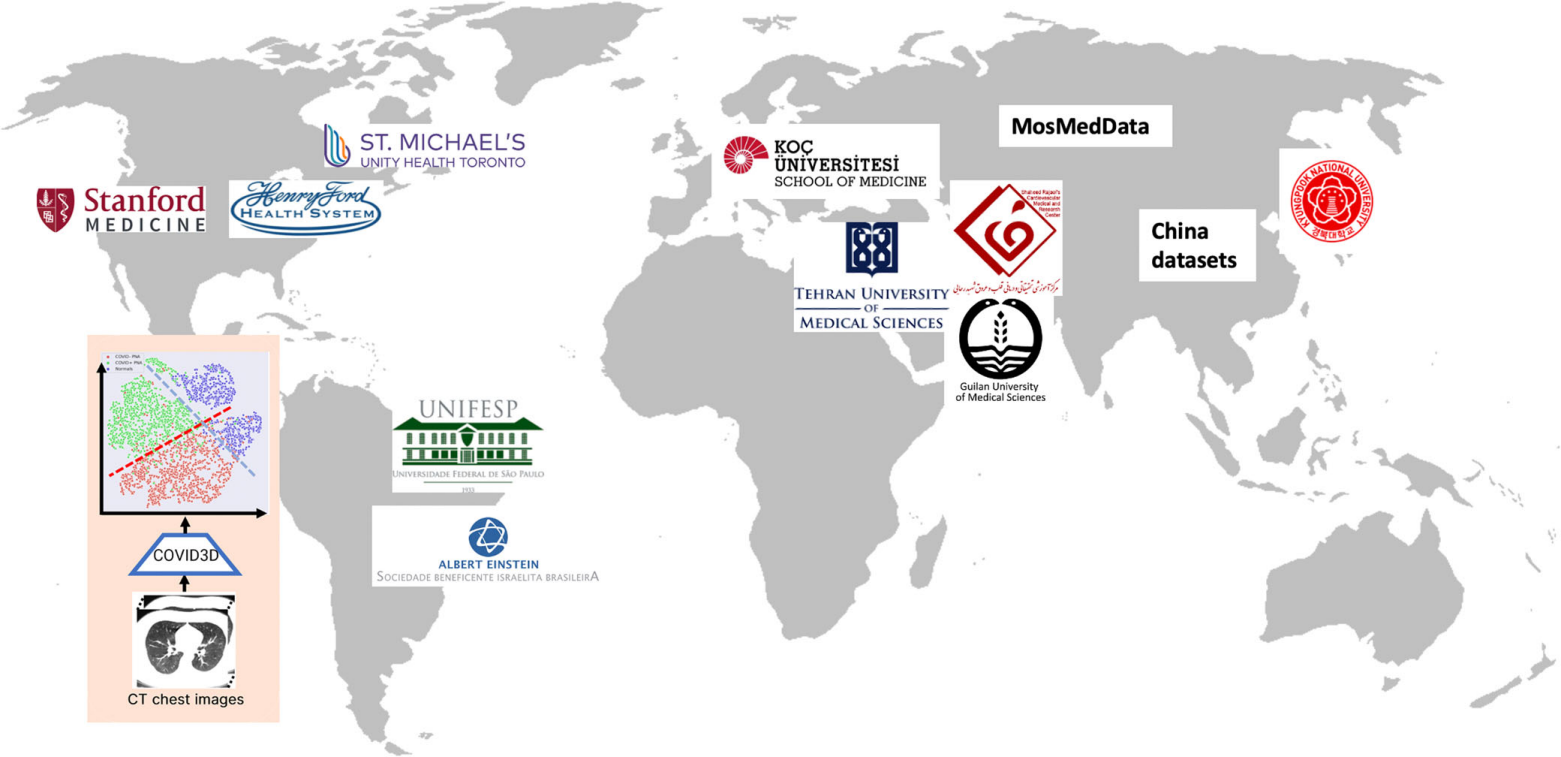
Institutions used in our study. Our AI model (DCD) captures the diversity of patients, labels, and scanners from around the world. Permission was sought and granted by all relevant institutions to use their logos.

**Fig. 2:**
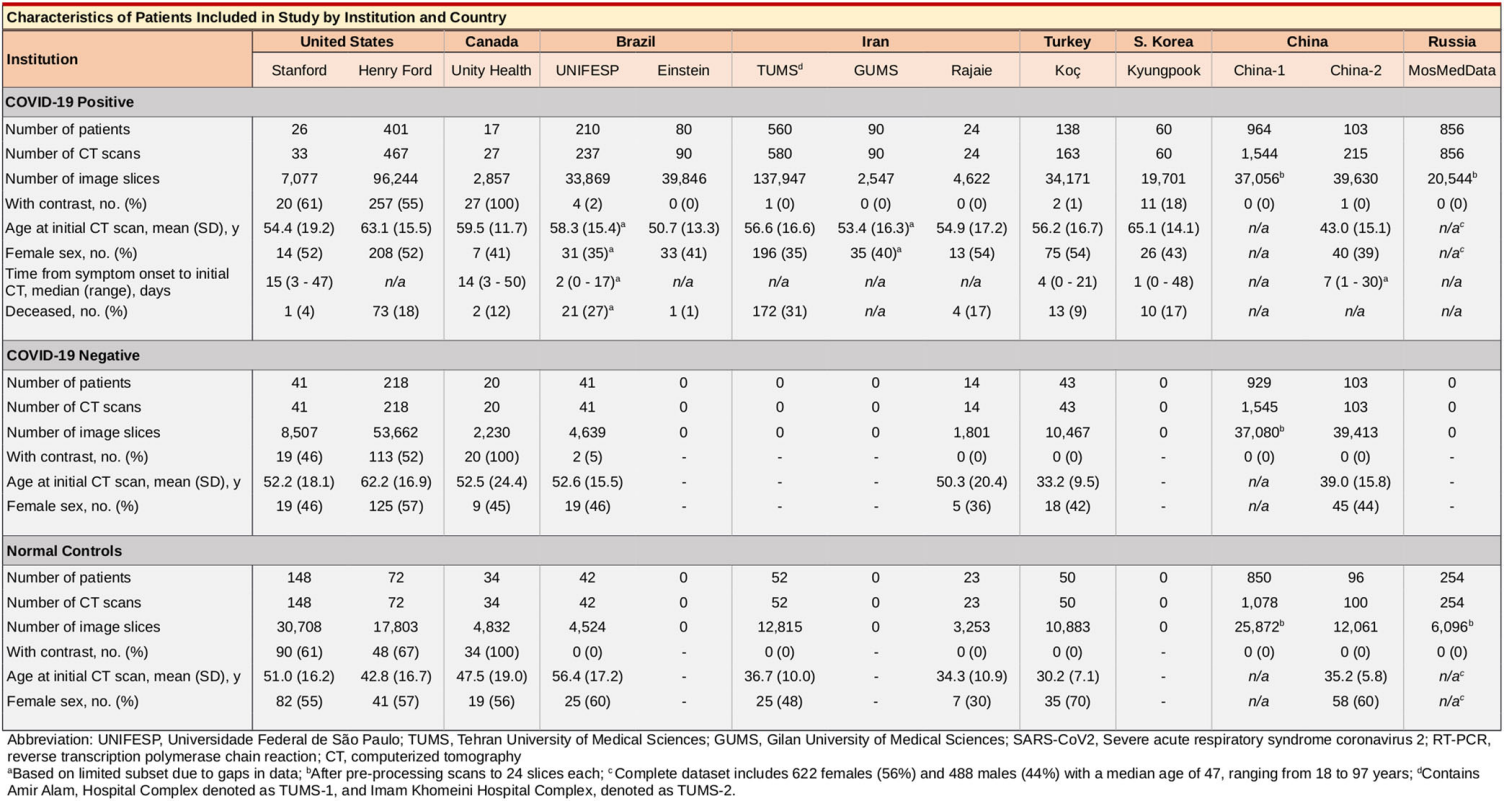
Characteristics of patients by institution and country. This table summarizes the patient demographics used in our study.

As the pandemic expands to global regions with limited access to nucleic acid detection kits, chest CT may play a greater diagnostic role for COVID-19 and disease monitoring, highlighting a need for automated or quantitative analytics. Recently, studies have reported success in deep learning methods with CT slices as inputs for COVID-19 classification^16–18^ or segmentation outputs to quantitate lung opacification and correlate disease severity^19^. Machine learning can capitalize on large-scale, high-dimensional image data and offers the opportunity to optimize a framework for COVID-19 evaluation, including prognostic models that stratify risk groups. This study goal was to develop Deep COVID DeteCT or DCD, a 27-layer 3D model, which (1) classifies COVID-19 pneumonia (COVID+) from non-COVID-19 (COVID−) pneumonia and normal lung, and (2) predicts disease course using chest CT. To the best of our knowledge, our study investigates one of the largest and most diverse patient population, and compares and discusses differences in performance across 13 international sites. The study sites are shown in Figs. 1, 2. Patient characteristics for each site is shown in Fig. 2.

- We designed a simple-to-use model (DCD) for classification trained and evaluated across 13 diverse sites from around the world.
- We investigate generalizability across all 13 sites, and discuss the contribution of participants outside of China on model performance.
- We track the disease course of COVID+ confirmed patients by using DCD features over time.

**Fig. 3:**
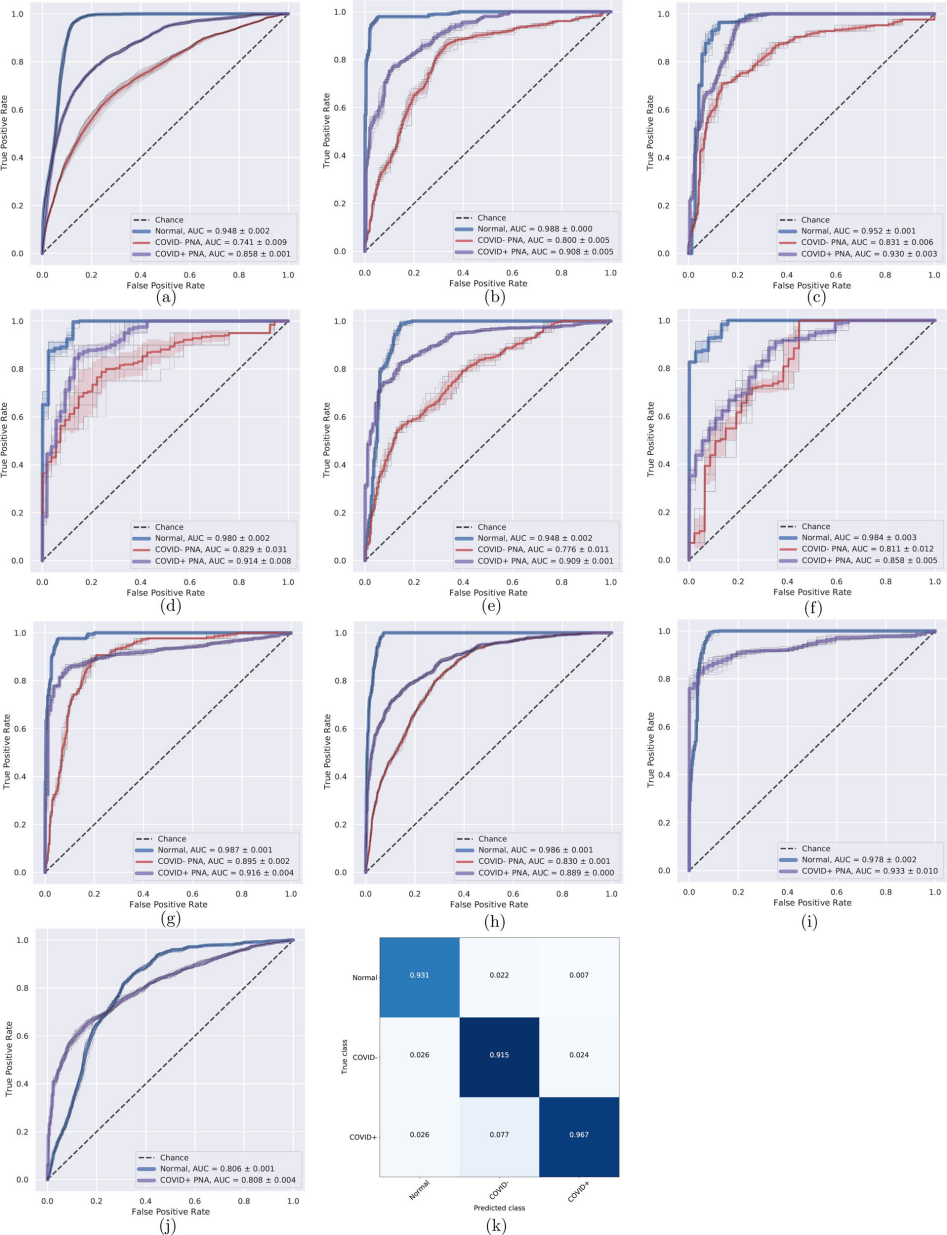
Receiver operating characteristics (ROC) curves and area under the curve (AUC) for different external test sites. The sites are: China-1 (**a**), China-2 (**b**), Stanford (**c**), Unity Health (**d**), Koç (**e**), Rajaie (**f**), UNIFESP (**g**), Henry Ford (**h**), TUMS-1 (**i**), MosMedData (**j**). ROC curves were not plotted for sites with imbalanced data: Kyungpook, GUMS, and TUMS-2. The confusion matrix for our model trained on all sites and finetuned on 20% of site China-1 (15% train, 5% validation) and evaluated on the remaining 80% of China-1 (**k**).

## Results

### A deep 3D model for classification using 13 international institutions

In task 1, we report high accuracies and Area under the Curve (AUC) in Table 1 with Receiver Operating Characteristics (ROC) curves shown in Fig. 3. Table 1 shows multiple training, validation, and test configurations. For example, with test site ID 9, the model is trained for 20 epochs and internally validated on all sites except Henry Ford, GUMS, and TUMS-2. After validation, the model is evaluated on the hold-out test site, Henry Ford. Our volumetric-based approach is also far superior to a 2D approach using a ResNet-50 pretrained on ImageNet, which yielded accuracies that were consistently lower. We also test the robustness of our models across variations of CT image windows for bone, soft tissue, and lung by changing pixel value thresholds during test-time. We evaluate DCD with variations in pixel threshold values in order to simulate the effect of sampling CT images at slightly different windows. In our ROC curves (Fig. 3), we plot the individual ROC curves and an averaged ROC curve with ±1 std. deviation error. This experiment is necessary to ensure that our model performance is robust and reproducible across a large diversity of scans. We ensured that slight variations in the window parameter leads to only modest and graceful degradation in ROC performance.

**Table 1:** Performance on all test sites.

Test site ID	Institution	Train/Val. sites	Normals AUC	COVID− PNA AUC	COVID+ AUC	Accuracy
0	China-1	1,..,11	0.948	0.741	0.858	0.707
1	China-2	0,2,...,11	0.988	0.80	0.908	0.789
2	Kyungpook	0,1,3,..,11	N/A	N/A	N/A	0.921
3	Stanford	0,..,2,4,..,11	0.952	0.831	0.93	0.804
4	Unity Health	0,..,3,5,..,11	0.98	0.829	0.914	0.775
5	Koç	0,..,4,6,..,11	0.948	0.776	0.909	0.779
6	Rajaie	0,..,5,7,..,11	0.984	0.811	0.858	0.767
7	Einstein	0,..,6,8,..,11	N/A	N/A	N/A	0.915
8	UNIFESP	0,..,7,9,..,11	0.987	0.895	0.916	0.828
9	Henry Ford	0,..,8,10,..,11	0.986	0.830	0.889	0.76
10	TUMS-1	0,..,9,11	0.978	N/A	0.933	0.881
11	MosMedData	0,..,10	0.806	N/A	0.808	0.747
12	GUMS	0,..,11	N/A	N/A	N/A	0.944
13	TUMS-2	0,..,11	N/A	N/A	N/A	0.974

Entries with N/A are due to class imbalance.

**Fig. 4:**
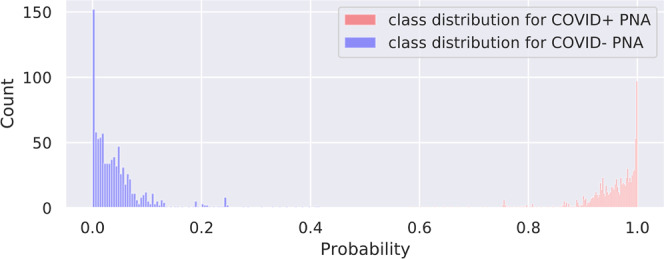
DCD predictions for Site 7. Histogram of model outputs on external hold-out test site 7 (Einstein) with only COVID+ cases.

**Fig. 5:**
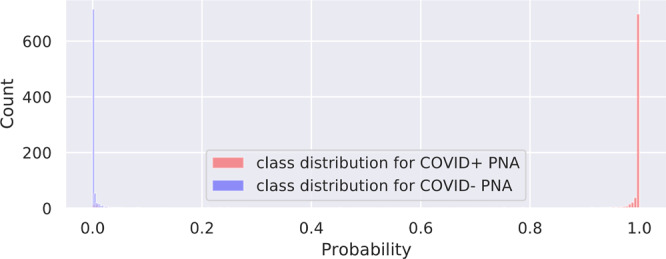
DCD predictions for Site 12. Histogram of model outputs on external hold-out test site 12 (GUMS) with only COVID+ cases.

**Fig. 6:**
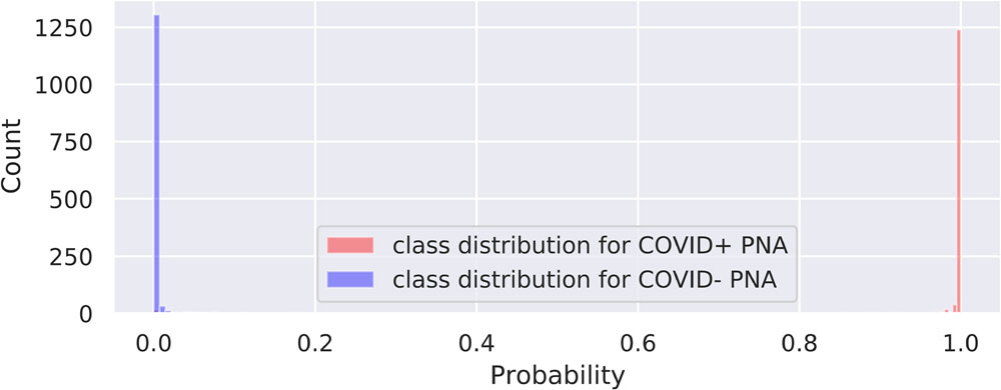
DCD predictions for Site 13. Histogram of model outputs on external hold-out test site 13 (TUMS-2) with only COVID+ cases.

**Fig. 7:**
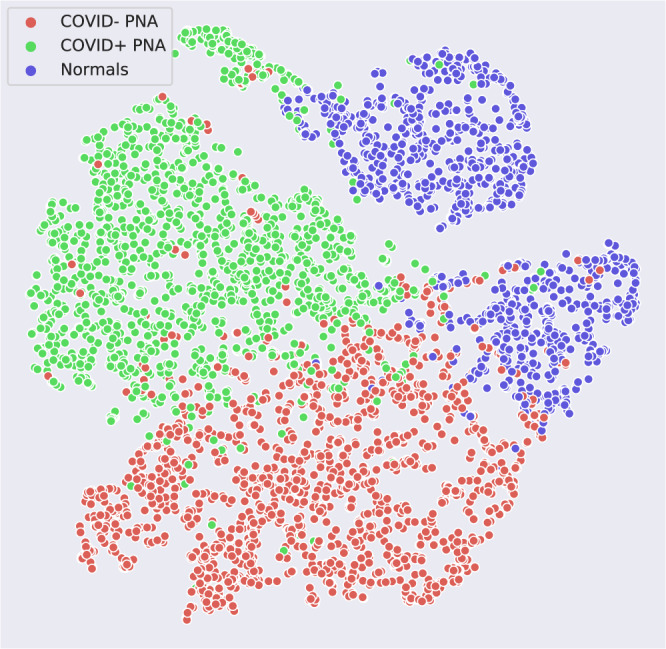
Two-dimensional manifold of features generated using t-distributed stochastic neighbor embedding (TSNE) on the DCD model. DCD evaluated on the test set of China-1 (80% of China-1). It was trained on sites 1 to 11 and finetuned on the training set of China-1 (20% of China-1).

**Fig. 8:**
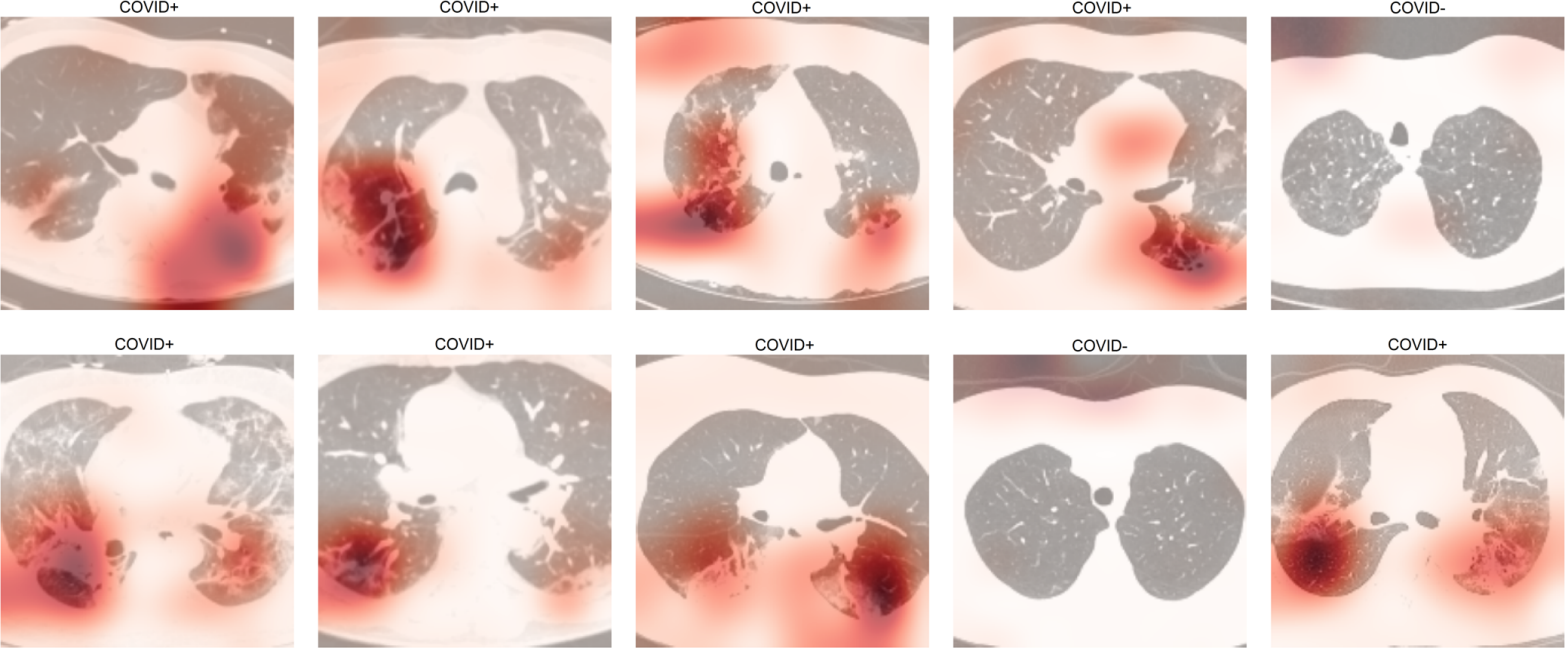
Grad-CAM over CT scans of COVID19+ and COVID19- pneumonia patients. On top right, DCD correctly diagnosed the scan of a COVID19- patient with PNA who showed signs of ground glass opacity.

**Fig. 9:**
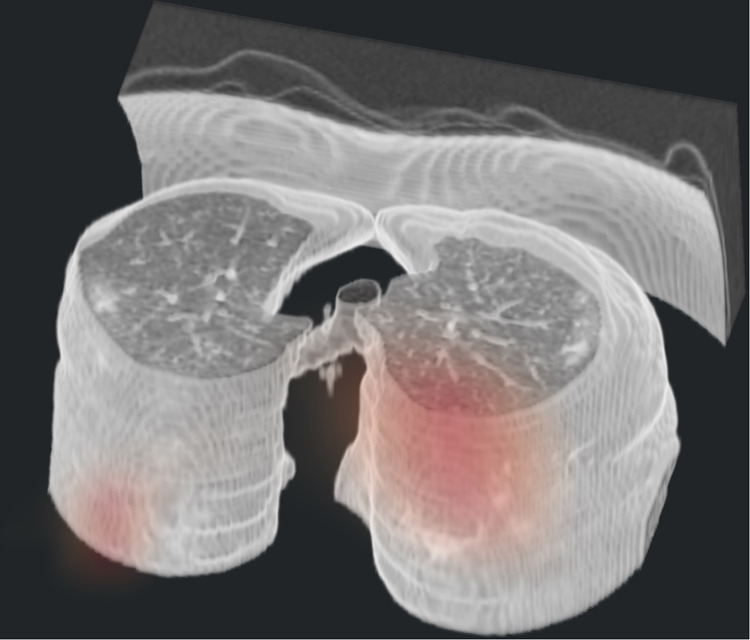
3D view of a model-generated 3D Grad-CAM superimposed on the CT of a COVID+ case with bilateral peripheral ground glass opacities and consolidation. The map was generated from only 1 forward and 1 backward pass of 1 example in the test set.

We investigate the contribution of non-China participants on performance. We train DCD on sites 0 and 1 only and test on non-China sites; we compare this strategy to one where we train DCD on all non-China sites. The results are shown in Table 2. We show that while AUCs were still very high except for two COVID− PNA AUCs, for most of the sites, the AUCs were significantly higher for the strategy that incorporated data from sites 2 to 11. ROC curves for these are shown in Supplementary Fig. 2. Furthermore, we investigate the effect of fine-tuning by (1) training a model on a set of sites, and (2) fine-tuning the model on a small subset of patients from the test site of interest. For example, we train DCD on sites 1 to 11 and fine-tune and internally validate for a maximum of 20 epochs on 20% of China-1 patients. The test accuracy on the remaining 80% of China-1 is 91.2%, whereas it is 71.6% without fine-tuning (70.7% on all China-1). Similarly, we train on sites 0 to 10 and fine-tune on 20% of MosMedData. Fine-tuning boosts performance from 73.2% to 80.6%. This is likely because the model learned to capture large variations in demographic and data collection practices. For example, COVID+ cases from Einstein site were of mild severity while those of TUMS-2 were severe; in fact, one-third of TUMS-2 COVID+ patients died. To illustrate this, we plot histograms of predictions for all COVID+ cases for Einstein (Fig. 4), GUMS (Fig. 5), and TUMS-2 (Fig. 6). COVID+ predictions from Einstein have lower confidence and higher variability than those of GUMS and TUMS-2. Next, we plot DCD features using t-distributed stochastic neighbor embedding (TSNE) (Fig. 7) to provide intuition of how the diagnosis predictions are arranged in a high-dimensional space. Finally, we used DCD to generate gradient-based heat maps^20^ (also known as Grad-CAM) on our external cohorts. In Fig. 8, we illustrate examples of where the DCD features activate strongly to key regions in the lungs. For example, in the COVID+ patients, we see almost all the ground glass opacity lighting up. In COVID− patients, COVID+ activation is limited. A 3D view of our model’s heatmap on a COVID+ patient is shown in Fig. 9.

**Table 2:** Performance of model trained on sites 0 and 1 versus a model trained on all sites.

Test Site	Institution	Train/Val. Sites	Normals AUC	COVID− PNA AUC	COVID+ AUC
3	Stanford	0,1	0.898	0.741	0.906
3	Stanford	0,..,2,4,..,11	0.952	0.831	0.93
4	Unity Health	0,1	0.844	0.603	0.859
4	Unity Health	0,..,3,5,..,11	0.98	0.829	0.914
5	Koç	0,1	0.875	0.351	0.822
5	Koç	0,..,4,6,..,11	0.948	0.776	0.909
6	Rajaie	0,1	0.903	0.775	0.724
6	Rajaie	0,..,5,7,..,11	0.984	0.811	0.858
8	UNIFESP	0,1	0.929	0.347	0.928
8	UNIFESP	0,..,7,9,..,11	0.987	0.895	0.916
9	Henry Ford	0,1	0.967	0.620	0.874
9	Henry Ford	0,..,8,10,..,11	0.986	0.830	0.889
10	TUMS-1	0,1	0.948	N/A	0.947
10	TUMS-1	0,..,9,11	0.978	N/A	0.933
11	MosMedData	0,1	0.636	N/A	0.604
11	MosMedData	0,..,10	0.806	N/A	0.808

### A method to describe disease trajectory and prognosis using learned features

**Fig. 10:**
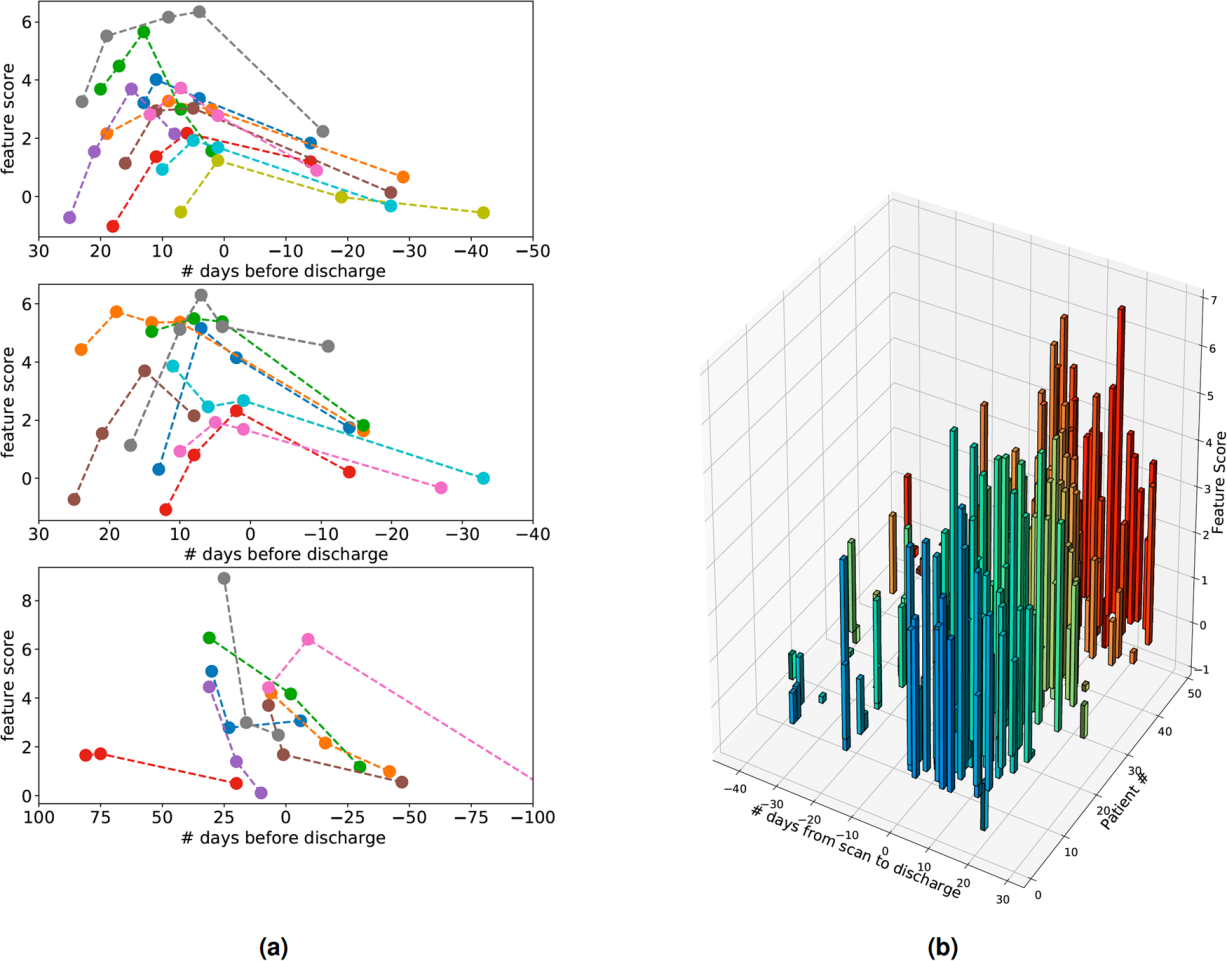
Features from DCD for the follow-up patients over time. Scans with high scores indicate high similarity to the COVID+ PNA population. Many patients show a representative feature trajectory with increasing COVID+ PNA intensity peaking near the time of discharge followed by a subsequent decrease after discharge. **a** Time-series of COVID-19 survivors with 2 or more follow-up scans, and (**b**) 3D plot of selected survivor scores that reveal similar trajectories.

In task 2, we deployed DCD on successive follow-up scans to measure DCD features over time. The goal was to track the progression of each patient’s individual disease status in an unsupervised manner. Higher feature scores for a given scan mean that the scan appears more similar to COVID+ population. In Fig. 10, we compute features denoted as *s*(*t*) for follow-up patients.

We show in Fig. 10(a) that many of the patients’ scores increased rapidly to a peak within 10 days. This was then followed by a gradual decline in COVID+ severity, which may indicate patient recovery. Even at and beyond the end of their hospitalization stay, many of the survivors’ lungs still contained features characteristic of COVID+ active disease. We theorize that we can use these features that map COVID+ severity over time to predict prognosis.

We quantify prognosis by the length of hospitalization (in days) measured from when the scan was imaged to time at discharge. A longer hospitalization window is indicative of worse prognosis. From our findings in Fig. 10, we expect that a large increase in DCD features over a short time window between 2 scans is indicative of a long hospitalization period and “high-risk” prognosis outcome. Similarly, a significant decrease in features over a short time indicates a low-risk prognosis. Features that grow in time but flatten out may also indicate low-risk. Furthermore, predicting prognosis is difficult with one scan alone, and knowing two scans may not be enough to tell when the patient’s feature score will peak. Two of these scenarios are shown in the Supplementary Fig. 3. Our intuition tells us that models trained to predict prognosis on hospitalization times can do better by looking at many sequential scans than just one scan alone. In the following experiments, we compared the prognostic performance of two scans (one prior and one follow-up) to one scan alone.

**Fig. 11:**
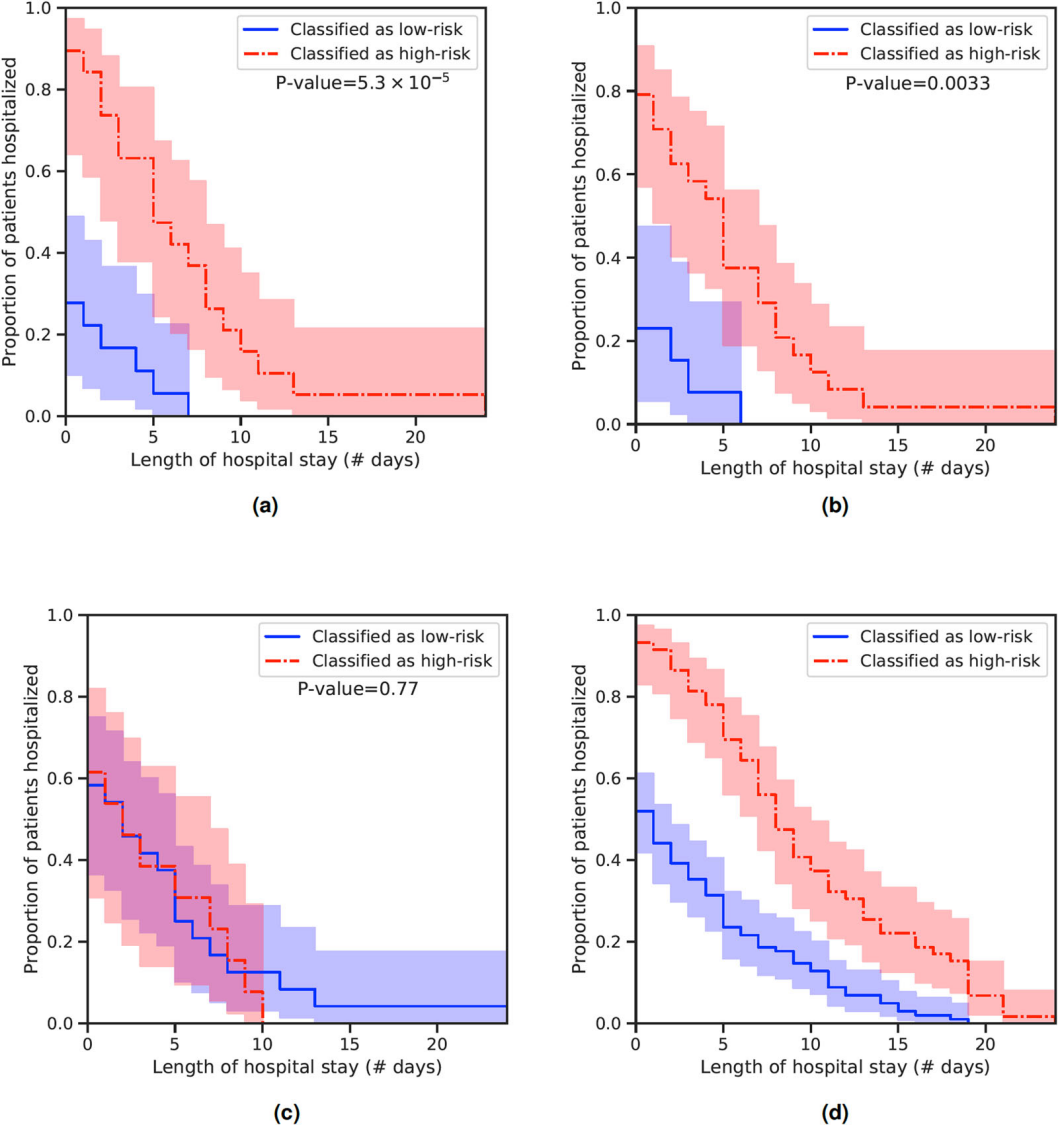
Kaplan–Meier plots on COVID disease course. Different configurations of DCD: (**a**) 1 prior +1 follow-up scans, (**b**) 1 scan only, (**c**) age and sex only, (**d**) 1 prior +1 follow-up scans (combines 5 validation folds in a 5-fold cross-validation experiment).

In Fig. 11 Kaplan–Meier (KM) are plotted on validation sets for different model configurations. The models stratify the patients into one of two groups: high-risk or low-risk. The first model (a) uses 2 sequential scans (one prior and one follow-up study) as inputs, while the second model (b) uses only one scan. We achieve greater separation using two sequential scans (log-rank *P* = 5.3 × 10^−5^). We achieve poor separation for a model trained using only age and sex as inputs (c). As an extension of (a), we perform 5-fold cross-validation and aggregate all 5 validation fold predictions in one KM plot. Finally, in order to account for any human-bias between discharge time and length of time between scans, we train a model using only the length of time as input (i.e., no images). This yields poor separation with *P* = 0.27.

**Fig. 12:**
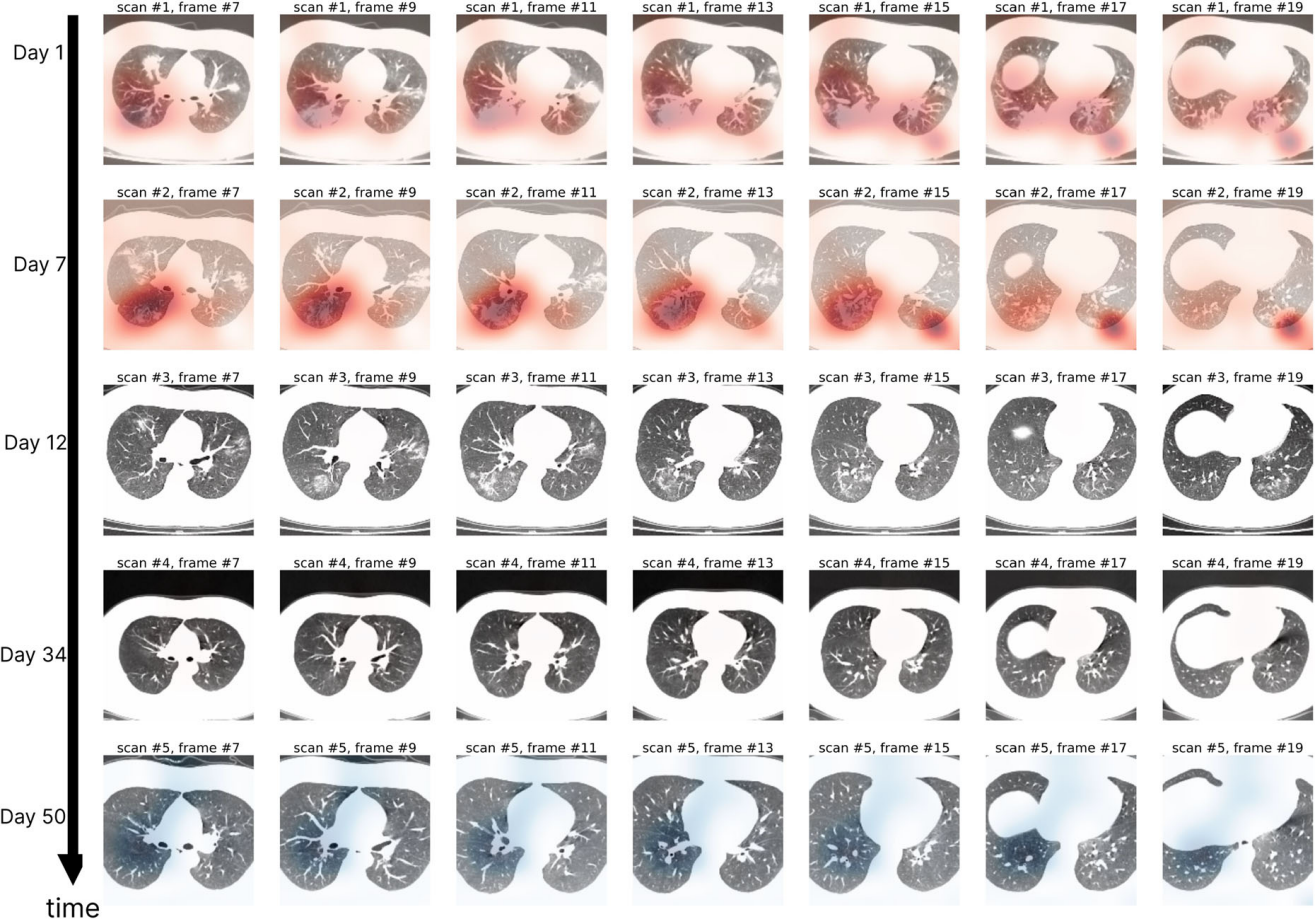
Case study using DCD on a follow-up patient (24 years old) with 5 scans. This patient was discharged on day 13. Modified gradient heat maps, *H*(*x*, *y*, *z*, *t*) for all pixel coordinates (*x*, *y*, *z*), are superimposed onto the original CT, and color-coded (red for *H*(*x*, *y*, *z*) > 0 and blue for *H*(*x*, *y*, *z*) < 0). The model was originally trained on task 1 and evaluated on these unseen examples. Severity predicted by DCD was highest on day 7 (as indicated by the visual difference between *H* on day 7 and day 1). On day 12, DCD’s *H*(*x*, *y*, *z*, *t* = 12) ≈ 0 indicates significant recovery.

To qualitatively assess the disease course of follow-up scans, we present heat maps that attend to regions in three dimensions in space and one in time. The Grad CAM^20^ of a 2D image, *H*(*x*, *y*), reveal pixels that activate strongly to the predicted class. *H*(*x*, *y*) is normalized throughout the entire (*x*, *y*)-plane from 0 to 1 for all pixel values (*x*, *y*). In our work, because we aim to track the disease trajectory from one scan to the next, we choose not to normalize with respect to one scan alone. Instead, we multiply the gradient maps by the feature score computed in Fig. 10. We compute a new map, *H*(*x*, *y*, *z*, *t*), across both space and time. This does two things: (1) scales the scan’s attention map by the degree of COVID severity at time *t* relative to all time points, and (2) provides direction information for when the CT becomes less similar to COVID+ data distribution. In Fig. 12, we plot *H*(*x*, *y*, *z*, *t*) on a 24 year old patient who had 5 scans in the course of 50 days. On day 13, the patient was discharged. The feature score *s*(*t* = 12) at day 12’s scan is almost 0, which is why *H*(*x*, *y*, *z*, *t* = 12) ≈ 0.

## Discussion

To the best of our knowledge, our study investigates one of the largest and most diverse patient population confirmed to have COVID-19 by RT-PCR tests and include 8 countries (Iran, Turkey, China, South Korea, USA, Canada, Brazil, Russia) with diverse geographic, genetic, racial, and ethnic backgrounds. We also include urban (e.g., Detroit) and nonurban (e.g., Palo Alto) centers. Some hospitals are specialty clinics, such as Rajaie Cardiovascular Institute in Iran, where majority are cardiac patients with clinical symptoms that mimic COVID-19 (e.g., shortness of breath) or have high-risk factors with a lower threshold for COVID-19 workup or CT screening, while others represent referral centers that treat complex medical conditions, or a combination of outpatients and inpatients (e.g., Unity Health-Toronto) admitted directly from active COVID assessment centers. Our image dataset is also diverse, acquired from multiple vendors (e.g., NeuViz, Siemens, GE, Philips, Toshiba) and with heterogeneous imaging protocols, that include contrast and non-contrast scans.

While many published AI works describe model performance, many models are learned on homogeneous data sources and thus raise questions of robustness and generalizability. Recognizing such general limitation, one recent study^18^ report model performance on COVID-19 data from China, Japan, and Italy. While the results are promising with high prediction accuracy (except for low accuracy in one Milan cohort), their controls consisted entirely of patients from the United States, which could raise concerns of bias. Notably, in our study, we find that contrast-enhanced CT is used widely in North America (California, Michigan, Toronto), whereas other international centers predominantly do not use contrast. This might reflect differences in clinical practice, where a CT may serve as a screening/diagnostic tool for COVID-19 (noncontrast CT) versus CT use to either problem-solve or evaluate other diseases (e.g., pulmonary embolism) associated with COVID-19. Using 13 international cohorts, we report high and robust accuracies and AUCs across all external test sites in Table 1.

**Fig. 13:**
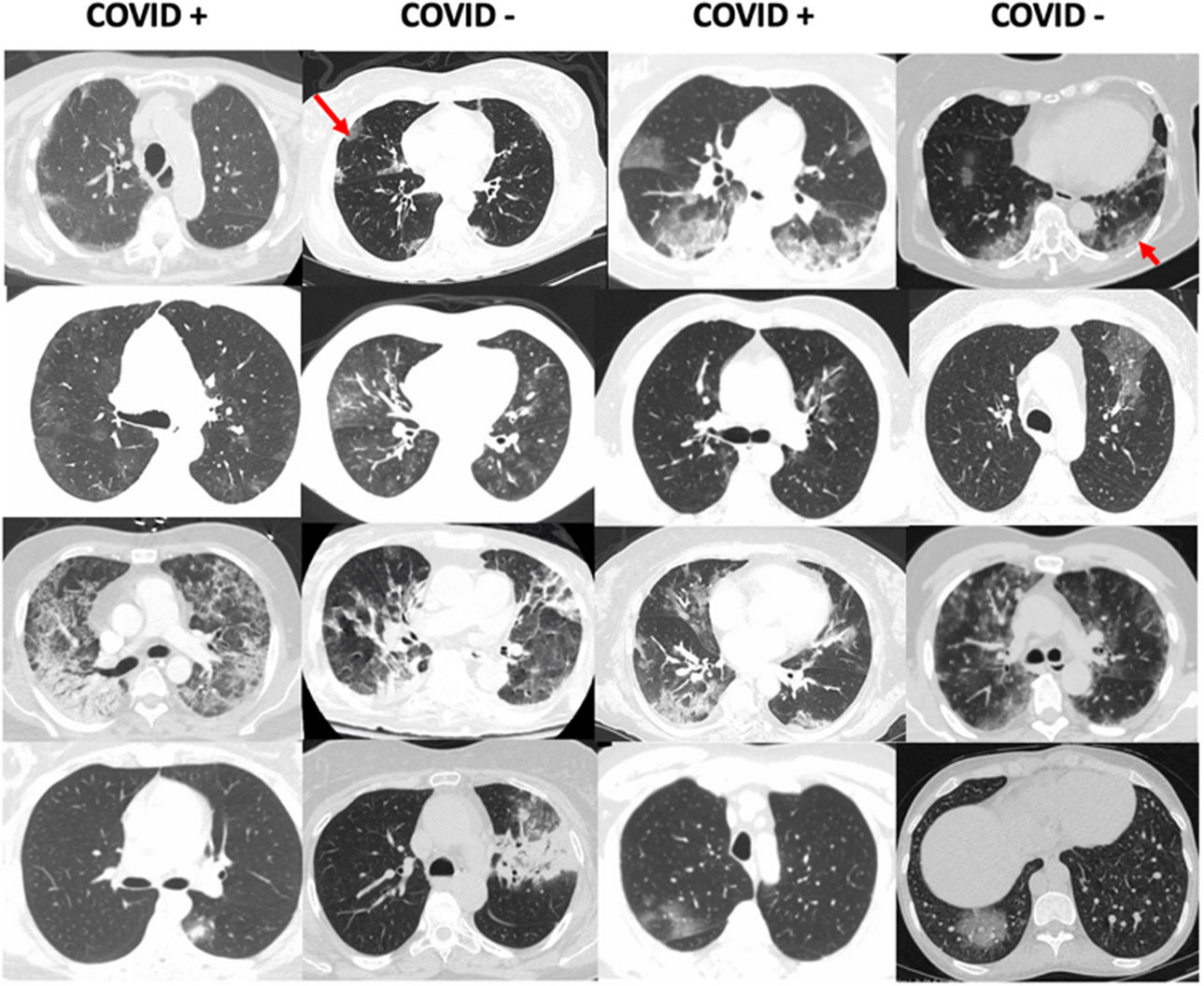
Examples of COVID− PNA patients in our study that show heterogeneous features, some that are similar to COVID+ PNA. For example, two COVID− patients with influenza PNA (red arrows), including H1N1 (long red arrow), show peripheral ground glass opacities similar to COVID+.

Unlike prior published AI works that combine lung segmentation and predictions^17,18,21–23^, we report use of a simple 3D model that uses whole CT chest that might facilitate clinical translation. Furthermore, while prior studies use human visual inspection ^23^, software-based segmentation for scoring disease severity^22,24,25^, we leverage learned features to conduct both supervised and unsupervised learning. Prior chest CT studies have shown characteristic COVID+ patterns, such as peripheral ground glass opacities that are often bilateral, peripheral with contiguous and multi-lobar extensions depending on disease severity^26–28^. COVID+ can also be present with dynamic features that might reflect disease evolution over time and recovery^19,29,30^. COVID+ features can overlap with those of other infections, inhalation injury, or drug toxicities. Figure 13 illustrates potential challenges in differentiating COVID+ versus COVID− pneumonia. Furthermore, many AI papers in COVID+ pneumonia detection have used either individual 2D slices or combined 2D CNN features to form 2.5D models^16,31–33^. One drawback is that using a 2D-only representation, such models could distort locality information in the sagittal direction.

Further, we show that DCD could identify features relevant to clinical outcome. We observe a distinct curve of features over time that is characteristic among almost all follow-ups (Fig. 10). We then apply supervision by fine-tuning DCD on hospitalization times. In this experiment, we show KM curves of two predicted populations deemed as high-risk or low-risk with hospitalization rates. Separation was largest in our model when presented with paired, temporally adjacent scans as opposed to one scan alone.

There are some limitations to our study. First, larger sample size is always desirable. Nevertheless, we demonstrate model generalizability in 13 sites and RT-PCR-positive 3529 unique patients affected with COVID-19. Creating any prognostic model has inherent challenges such as the existence of many complex clinical variables. We do not examine the effects of different therapies, which is beyond the scope of this study.

In conclusion, we present DCD, a single 3D model that diagnoses and tracks disease course over hospitalization without the aid of complex preprocessing. We leverage transfer learning from Kinetics video dataset for classification; we show robust generalizability across diverse international cohorts. We show indicative patterns in DCD features that correlate with the patient outcome. Finally, we show heat maps that highlight the visual progression of a patient’s disease trajectory over time.

## Methods

### Multi-center dataset

We conducted a multi-center retrospective study across 13 institutions including 2 COVID+ datasets from China^17^ and 1 from Russia^34^. Institutional review board (IRB) at participating hospitals approved this retrospective study with waiver of consent. Waiver of consent was granted by the IRB for the following reasons: (1) The research involves no more than minimal risk to the participants because it involves materials (data, documents, records) that have been or will be collected, and precautions will be taken to ensure that confidentiality is maintained, (2) the waiver will not adversely affect the rights and welfare of the participants because procedures are in place to protect confidentiality, and (3) information learned during the study will not affect the treatment of participants. Patient demographics are summarized in Fig. 2. The following represents the inclusion criteria: patients presented with clinical symptoms suspicious for COVID-19 pneumonia, obtained at least one confirmatory real time RT-PCR tests to determine COVID-19 status, and obtained diagnostic quality chest CT. For RT-PCR testing, samples of respiratory secretions from bronchoalveolar lavage, endotracheal aspirate, nasopharyngeal or oropharyngeal swabs were used. Scanner models and slice thicknesses for participating institutions are shown in Supplementary Fig. 1.

### Image preprocessing

All raw data from institutions except for China-1 and MosMedData were provided in Digital Imaging and Communications in Medicine (DICOM) format. The majority of the patient DICOMs contained dynamic range of pixel intensities consistent with that of a lung window. Patient DICOM series were collected and scaled to 256 × 256 pixels. DCD was designed to sample 24 planes evenly across the lung. To accommodate a large range of slice thickness (1 to 5-mm), DCD uniformly sub-samples across the sagittal plane until 24 approximately-equidistant slices are extracted per patient. For data augmentation during training, we also generate additional 24-plane samples by applying random jitter in the depth dimension. Finally, before each 24 × 256 × 256 image is fed into the model for training, we apply a clipping function that truncates all Hounsfield unit intensity values above a fixed pre-determined value. This was to ensure that large Hounsfield Unit values outside the lung (e.g., bone) does not saturate and overwhelm the signal from the lung. During training, we randomly cropped images to 240 × 240 and resized them back to 256 × 256, performed random flipping in the *x* and *y* directions, and applied random jitter in the depth dimension. During test-time, we measure ROC curves and AUCs across different clipping values (±6.25% jitter) to simulate the effect of sampling CT images at slightly different windows.

### DCD model training and evaluation

The DCD model consists of a 27-layer I3D-Inception feature extractor, 3D spatial average pooling, and 1 fully-connected layer. The Inception model^35^ was pretrained on Kinetics-600, a video dataset^36^.

In task 1, we designate one institution as the external hold-out test set while pooling (merging) the others into the training and and internal validation sets. The internal validation set is generated by randomly sampling 5% of the pooled data without replacement. Training consisted of minimizing cross-entropy loss with dropout and Adam optimization^37^ for 20 epochs. We evaluate on the internal validation set at every fifth of an epoch. After training, we select the trained weights that maximizes the validation accuracy to be used for evaluation on the test site. Future improvements such as stratified sampling and selection of models that maximizes the worst-case performance over all sites are topics of future research. In Table 1, we illustrate the performance on all test sites.

In classifiers trained under cross-entropy loss, the output is a set of prediction probabilities that correlate towards COVID disease severity. In task 2, we use the (pre-activation) logit, denoted as *s* throughout this paper, as the feature we use to track disease course. This feature can be interpreted as follows: if the feature score of a patient is *s*(*t* = 0) > 0 and, $\frac{{\rm{d}}s}{{\rm{d}}t}\,>\,{0}$, the sequence of scans is transforming to be more COVID-like; if $\frac{{\rm{d}}s}{{\rm{d}}t}\,<\,{0}$, it suggests that this patient is recovering. Using these features, we fine-tuned DCD, which was trained on sites 1 and 2 in classification task 1, to predict patient prognosis on follow-up scans. Due to the limited number of follow-ups, we split the scans on a per-patient basis into either training or validation sets; we also perform an additional 5-fold cross-validation procedure and aggregate predictions from each of the 5 validation folds. In order to predict prognosis using two consecutive scans, the DCD’s convolutional model backbone computed features for scans 1 and 2, and concatenated features were passed through two fully-connected layers. On the other hand, a model predicting prognosis using a single scan alone uses only the convolutional backbone and two fully-connected layers without concatenation. We quantify prognosis by the length of hospitalization time (in days) measured from when the scan was imaged to time at discharge. A longer hospitalization time is indicative of worse prognosis. Rather than predicting the time using regression, we treat this problem as a binary classification problem to classify whether the patient will stay hospitalized for longer than 7 days (median) given the presenting scan at any given time of the patient’s disease course. We define 7 days or longer to indicate a subjective “high-risk” prognosis and below 7 days to indicate a “low-risk” status. We perform classification instead of regression to respect the fact that hospitalization times are inherently noisy. For instance, a model learning to achieve 0 root-mean-square error on patients whose discharge was delayed by non-medical reasons is counterproductive. Our model uses binary cross-entropy loss instead of Cox proportional-hazards loss^38^ since (1) we designed this task as a classification problem to compare and interpret easily to radiologists and (2) asking radiologists to predict the number of days is not common clinical practice.

### Reporting summary

Further information on research design is available in the Nature Research Reporting Summary linked to this article.

## Supplementary information

Reporting Summary

Supplemental Figures

## Data Availability

Not all the datasets generated and analyzed during the study are currently publicly available. Datasets from Stanford, Koç, Unity Health, and UNIFESP have been submitted to the Radiological Society North America for public release: RSNA International COVID-19 Open Radiology Database (RICORD)^39^, and will be publicly available in the near future.

## Code availability

We plan to open-source code at a future date with incremental public updates as we obtain more results and data. Please check in https://github.com/edhlee/Deep-COVID-DeteCT for updates.
