# Supplementary material for: Deep COVID DeteCT: an international experience on COVID-19 lung detection and prognosis using chest CT
Source: NPJ Digit Med. 2021 Jan 29;4:11. doi: 10.1038/s41746-020-00369-1 (PMC7846563; doi:10.1038/s41746-020-00369-1)
Supplement: Supplementary file 2 — Supplemental Figures [file 41746_2020_369_MOESM2_ESM.pdf]

Supplementary Information

CT Scanner Models, Products, and Specifications by Institution										
**Institution**	**Stanford**	**Henry Ford**	**Unity Health**	**UNIFESP**	**Einstein**	**TUMS**	**GUMS**	**Rajaie**	**Koç**	**Kyungpook**
**Slice Thickness**	1mm	1.25mm<br>1.4mm<br>2mm	3mm	3mm	1.0mm	0.75mm<br>1.5mm<br>2.5mm	1.2mm	1.5mm	1mm<br>1.5mm	1mm<br>1.25mm<br>5mm
**Image Matrix**	512 x 512	512 x 512	512 x 512	512 x 512	512 x 512	512 x 512	512 x 512	512 x 512	512 x 512	512 x 512
**Models**	GE LightSpeed VCT<br><br>Siemens SOMATOM Definition Flash<br><br>Siemens SOMATOM Force	GE LightSpeed VCT<br><br>GE Optima CT660<br><br>Philips Brilliance 64<br><br>Toshiba Aquilion	GE LightSpeed VCT<br><br>GE Optima CT660<br><br>GE Revolution CT	Philips Brilliance 64	Siemens SOMATOM Definition AS<br><br>Siemens Biograph 40<br><br>Toshiba Aquilion	Siemens SOMATOM Emotion 6<br><br>Siemens SOMATOM Emotion 16	GE HiSpeed NX/I Dual	Siemens SOMATOM Scope	GE LightSpeed Pro 16<br><br>GE Revolution CT<br><br>Philips Brilliance 64<br><br>Siemens SOMATOM Definition AS<br><br>Siemens SOMATOM Definition Flash	GE Optima CT660<br><br>Siemens SOMATOM Definition AS<br><br>Siemens SOMATOM Perspective

Supplementary Figure 1. Scanner models and slice thicknesses by institution.

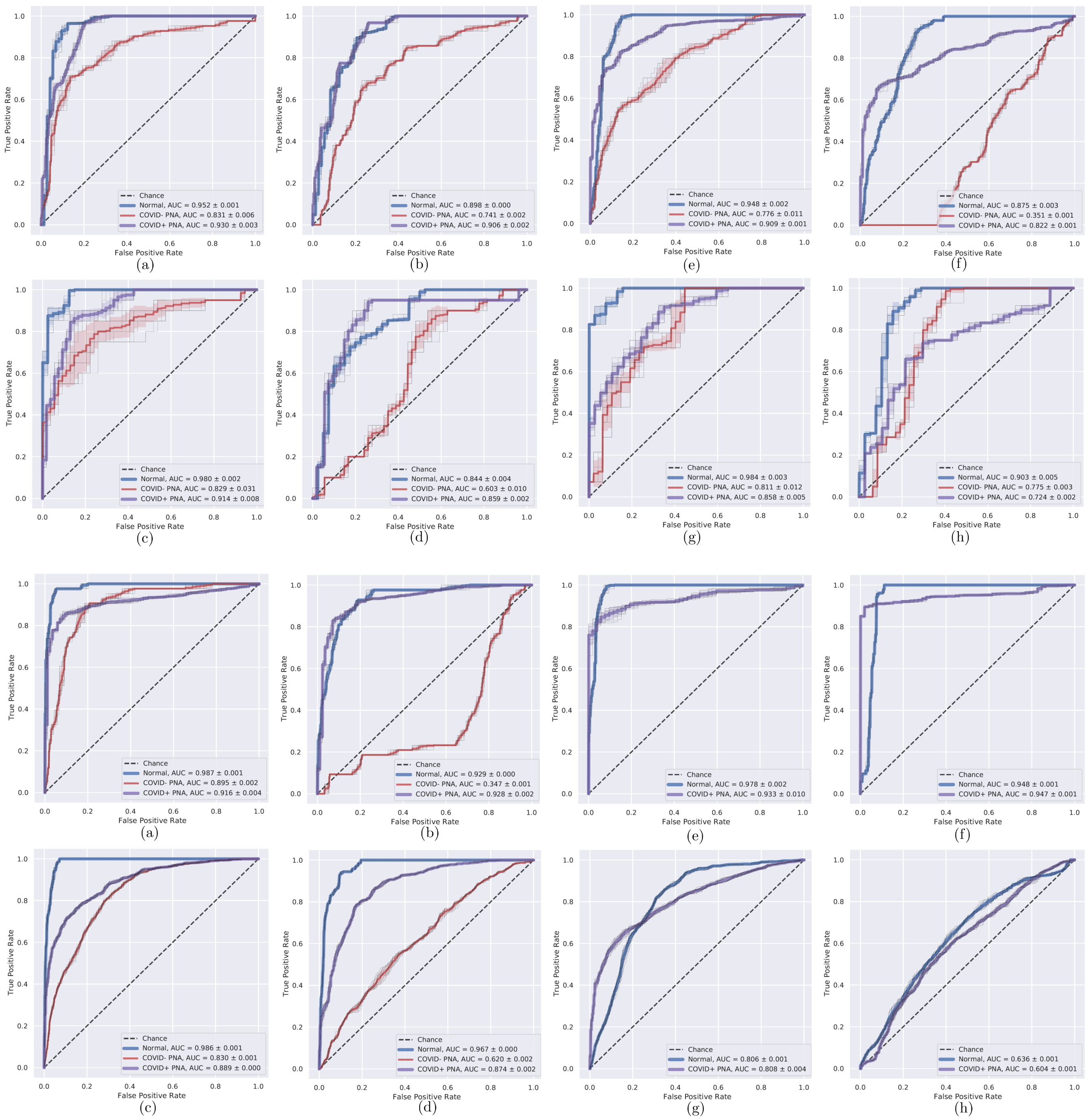

Supplementary Figure 2. ROC curves for Table 2 of the manuscript comparing performance with and without training on sites 2 to 11. Sites are 3 (a, b), 4 (c,d), 5 (e,f), 6 (g,h) as shown on the top half, and 8 (a,b), 9 (c,d), 10 (e,f), 11 (g,h) on the bottom half.

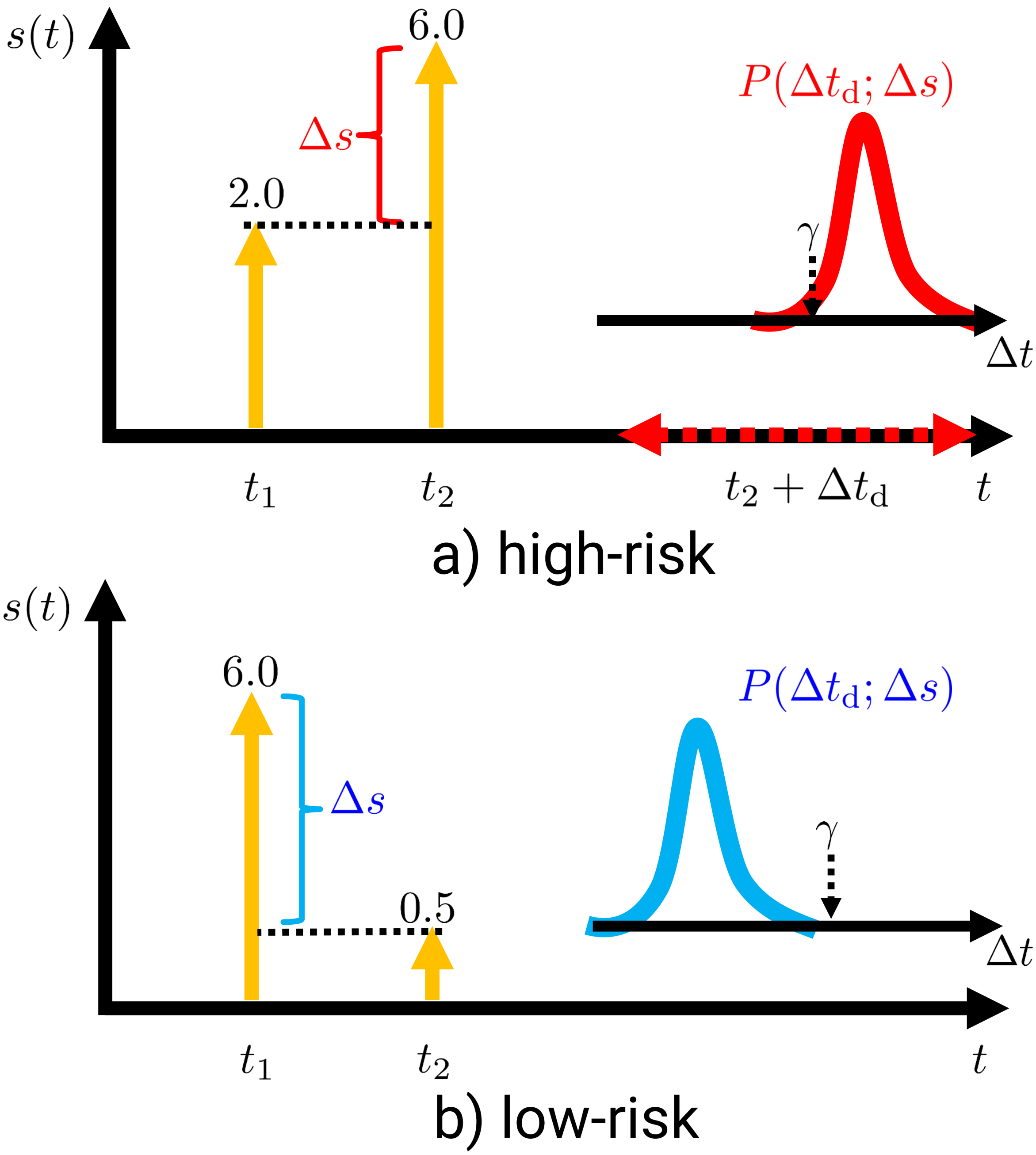

Supplementary Figure 3. Two scenarios of using DCD features to predict prognosis: patient A (top, a) is scanned on day t_1 and t_2. DCD predicts COVID scores of $s(t_1) = 2$ and $s(t_2) = 6$, indicating a significant rise in COVID-like features. If scan dates are close apart, then it is reasonable to say that patient A's prognosis is "high-risk" and may remain hospitalized for longer than average $\Delta t_d > \gamma$. Conversely, patient B's prognosis is classified as "low-risk". P represents the probability density function of the time-to-discharge parameter Δt_d.
